# Integrated Analysis of ceRNA Network to Reveal Potential Prognostic Biomarkers for Glioblastoma

**DOI:** 10.3389/fgene.2021.803257

**Published:** 2022-02-14

**Authors:** Ruifei Liu, Zhengzheng Gao, Qiwei Li, Qiang Fu, Dongwei Han, Jixi Wang, Ji Li, Ying Guo, Yuchen Shi

**Affiliations:** ^1^ Second Affiliated Hospital, Heilongjiang University of Chinese Medicine, Harbin, China; ^2^ College of Basic Medicine, Inner Mongolia Medical University, Hohhot, China; ^3^ Heilongjiang University of Chinese Medicine, Harbin, China; ^4^ Jiaxing University, Jiaxing, China; ^5^ First Affiliated Hospital of Heilongjiang University of Chinese Medicine, Harbin, China

**Keywords:** glioblastoma, lncRNA, ceRNA, network, prognostic biomarker

## Abstract

Glioblastoma (GBM), originating in the brain, is a universally aggressive malignant tumor with a particularly poor prognosis. Therefore, insight into the critical role of underlying genetic mechanisms is essential to developing new therapeutic approaches. This study aims to identify potential markers with clinical and prognostic significance in GBM. To this end, increasing numbers of differentially expressed RNA have been identified used to construct competitive endogenous RNA networks for prognostic analysis via comparison and analysis of RNA expression levels of tumor and normal tissues in glioblastoma. This analysis demonstrated that the RNA expression patterns of normal and tumor samples were significantly different. Thus, the resulting differentially expressed RNAs were used to construct competitive endogenous RNA (competing endogenous RNA, ceRNA) networks. The functional enrichment indicated mRNAs in the network are critically involved in a variety of biological functions. Additionally, the prognostic analysis suggested 27 lncRNAs, including LOXL1-AS1, AL356414.1, etc., were significantly associated with patient survival. Given the prognostic significance of these 27 lncRNAs in GBM, we sought to classify the samples. Importantly, Kaplan-Meier analysis revealed that survival times varied significantly among the different categories. Overall, these results identify that the candidate lncRNAs are potential prognostic markers of GBM and its corresponding mRNAs may be a potential target for therapy.

## Introduction

Long non-coding RNAs (lncRNAs), a series of transcript RNAs longer than 200 nucleotides, plays a very crucial role in biological processes, such as cell proliferation, cell apoptosis, and cell cycle regulation (Zhang et al., 2020). Accumulating studies reported that lncRNA can be involved in the regulation of competitive endogenous RNA (ceRNAs) to communicate with other RNA transcripts (Calin et al., 2007; Arvey et al., 2010; Ebert and Sharp, 2010). LncRNA can function as an endogenous molecular sponge, indirectly regulating downstream mRNA expression levels by having shared microRNA response elements with reverse complementary binding seed regions competitively binding to miRNA, and subsequently involved in cancer development (Bai et al., 2019; Sun et al., 2020). In other words, lncRNA competes with miRNA target genes for miRNA molecules by sharing a common miRNA binding site with mRNA. It has been documented that ceRNAs play a regulatory role in gene expression and is involved in the pathogenesis of diseases such as cancer (Tay et al., 2011). A growing body of evidence clarifies that molecular networks play an important role in a variety of human diseases (Silverman et al., 2020). Accordingly, it is valuable to dissect the ceRNA network for understanding the underlying molecular mechanisms of cancer development.

Glioblastoma (GBM), one of the most fatal and aggressive forms of brain tumors, is a prevalent malignant tumor that originates in the brain, currently accounting for more than half of all gliomas (Liang et al., 2005). GBM is characterized by its high invasiveness, poor clinical prognosis, and high mortality rates. Current therapeutic approaches include focal radiotherapy, chemotherapeutics, and surgical resection. The 5-years survival rate is less than 3% (De Leo et al., 2020). Over the past few years, little progress has been made in determining methods to predict which patients will better receive the current standards of care (Johnson et al., 2020). Although survival has improved with the optimization of treatment strategies, GBM prognosis remains poor (Wen and Kesari, 2008; Yuan et al., 2015). Consequently, investigating potential genetic mechanisms of GBM is of great significance. The development of alternative and suitable biomarkers to effectively diagnose and treat GBM remains one of the most pressing challenges in cancer therapy (Aldape et al., 2015; Zhou et al., 2019). Identification of prognostic markers of GBM also contributes to comprehending the mechanisms of metastasis, which may lead to the discovery of novel therapeutic targets. The exploration of ceRNA networks in GBM may provide new insight into understanding the biological mechanisms of the disease.

In this study, glioblastoma-specific ceRNA networks were constructed based on differentially expressed genes. In addition, we further derived and characterized the lncRNAs that were significantly associated with survival in the network, classified the samples based on the screened lncRNAs. We observed the significant differences in survival time among the types of samples, which could shed light on that the lncRNAs we screened are potential prognostic markers of GBM and its corresponding mRNA may be a potential target for therapy.

## Materials and Methods

### Acquisition of Glioblastoma Transcriptome and Clinical Data

We obtained the transcriptome expression profile in glioblastoma with 154 tumor samples and 5 normal samples *via* The Cancer Genome Atlas (TCGA) database (https://tcga-data.nci.nih.gov/tcga). Moreover, we also retrieved the demographic information (age, gender, race and so on) and survival endpoint (vital status, days to death and days to last follow-up) of each patient.

### Interactions of ceRNA

StarBasev2.0 (http://starbase.sysu.edu.cn/index.php) database is an open-source platform for decoding miRNA–ceRNA, miRNA–ncRNA, and protein–RNA interaction networks, stored the lncRNA related ceRNA interactions identified using hypergeometric tests (Li et al., 2014). The hypergeometric test (Sumazin et al., 2011) is executed for each ceRNA pair separately, which is defined by four parameters: 1) N is the total number of miRNAs used to predict targets; 2) K is the number of miRNAs that interact with the chosen gene of interest; 3) n is the number of miRNAs that interact with the candidate ceRNA of the chosen gene; and 4) c is the common miRNA number between these two genes. The test calculates the *p*-value by using the following formula:

$$P=\overset{\text{min}\left(K,n\right)}{\underset{i=c}{\sum }}\frac{\left(\begin{array}{c} K\\ i \end{array}\right)\left(\begin{array}{c} N-K\\ n-i \end{array}\right)}{\left(\begin{array}{c} N\\ n \end{array}\right)}$$ (1)

Multiple miRNAs belonging to the same family were every miRNA family only once, even if it had multiple binding sites at the same 3′-UTR of protein coding genes or transcript of non-coding genes. All *p*-values were subject to false discovery rate (FDR) correction.

In this study, starBase was utilized to downloaded and extracted the ceRNA-ceRNA interactions of lncRNA-mRNA.

### Associations Between LncRNA and Cancer

We downloaded the relationships between lncRNA and cancer from the Lnc2Cancer 3.0 (Sumazin et al., 2011) (http://www.bio-bigdata.net/lnc2cancer/) database, which contains the associations verified by the literature of 2,659 human lncRNAs and 216 cancer subtypes.

### Identification of Differentially Expressed LncRNAs and mRNAs

First, we screened out genes that expressed less than 2 in 20 % of the samples. Next, compared to the normal group with the tumor group, the R software (version 3.6.3) and limma package in Bioconductor were used to detect the differentially expressed lncRNAs (DElncRNAs) and mRNAs (DEmRNAs). DElncRNAs and DEmRNAs were identified using the selection criteria of adjusted *p*-value (FDR) < 0.01 and FC > 2.0 or FC < 0.5 calculated by the T-test and fold change algorithm. Then, the differentially expressed lncRNAs and mRNAs meeting the criteria were displayed in volcano plots.

### Construction of Glioma-specific LncRNA-mRNA ceRNA Network

Ahead of analyzing the basic statistics, we downloaded information from starBase about the lncRNA-ceRNA interaction. All interactions are verified by the literature. The starBase database contains 83,916 lncRNA-ceRNA interactions, including 2,539 lncRNAs and 2079 mRNAs. After that, we mapped the DEmRNAs and DElncRNAs selected in the previous step to the lncRNA related ceRNA interactions. Subsequently, the interactions between DEmRNAs and DElncRNAs were singled out to construct a glioma-specific ceRNA regulatory network. Cytoscape (version 3.7.2) was used to visualize the ceRNA network.

### Functional Enrichment Analysis

Gene Ontology (GO) is a universal tool for defining the biological process (BP), cellular component (CC), and molecular function (MF) of numerous genes. Kyoto Encyclopedia of Genes and Genomes (KEGG) pathway is a database that contains multiple biological pathways for several organisms (Kanehisa et al., 2017). The enrichment analyses of mRNAs on the glioma-specific ceRNA network were performed using the clusterProfiler package in Bioconductor, and a *p*-value less than 0.05 was considered as statistically significant (Yu et al., 2012). Furthermore, we performed a KEGG pathway enrichment for mRNAs connected to each lncRNA. GO and pathway analysis provided a deep insight into the relations of functions or pathways and the primary roles of these genes.

### Survival Analysis

The Cox proportional hazards regression model has the function to process the truncated survival time while analyzing various variables with no requirement for the type of distribution of the survival function (Zhao et al., 2010). To assess the prognostic characteristics of all lncRNAs, the univariate Cox proportional hazards model was applied. We integrated all lncRNAs on the glioma-specific ceRNA network into the univariate Cox model to identify the lncRNAs significantly associated with survival. *p* values < 0.05 were regarded as significant.

### Prognostic Analysis

\K-means clustering algorithm was used to classify the samples into four groups based on lncRNA that was significantly related to survival and R package “factoextra” was adopted to visualize it. To further determine the prognostic characteristics of lncRNAs, after combining the overall survival of 154 patients with GBM, the survival curves of these samples with classification information were plotted by using the “survival” package in R based on Kaplan-Meier curve analysis. Log-rank *p* < 0.05 was considered significant.

## Result

### Identification of Differentially Expressed Genes in Glioblastoma

**FIGURE 1:**
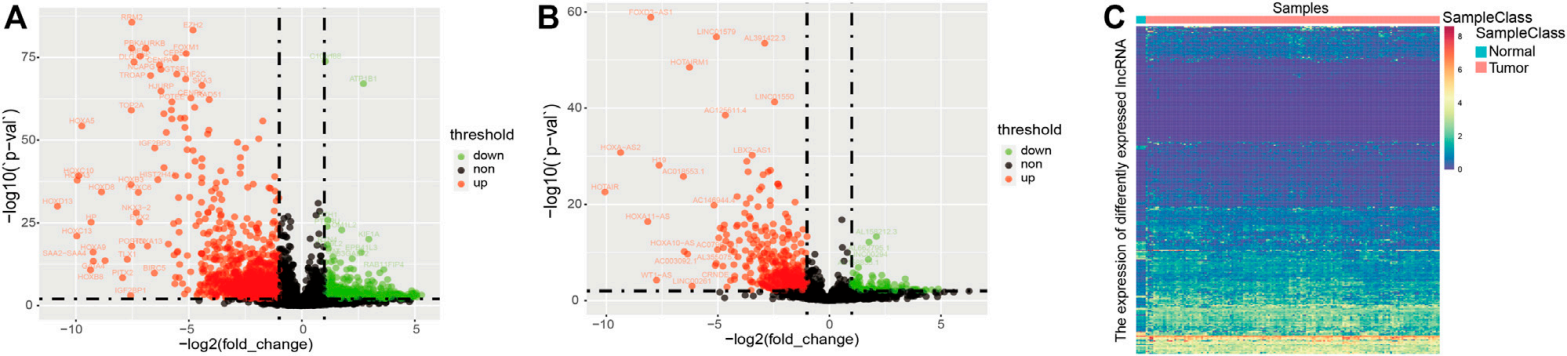
**(A)** Volcano map of the differential mRNA. **(B)** Volcano map of the differential lncRNA. **(C)** Heatmap plots of the differentially expressed lncRNAs between normal and cancer samples.

In order to better explore the differences between glioma patients and normal samples at the gene transcriptome level, based on the dataset of 5 normal samples of glioblastoma and 154 cancer samples derived from TCGA, we performed a differential expression analysis to identify significantly differentially expressed lncRNAs and mRNAs. Then, as shown in Figures 1A,B , we compared the tumor group with the normal group to visualize significantly differentially expressed lncRNAs and mRNAs using volcano maps. Finally 2,326 DElncRNAs (Figure 1A, B) and 8,304 DEmRNAs were identified (Figure 1C).

### Dissecting ceRNA Network Reveals lncRNA Functions

**FIGURE 2:**
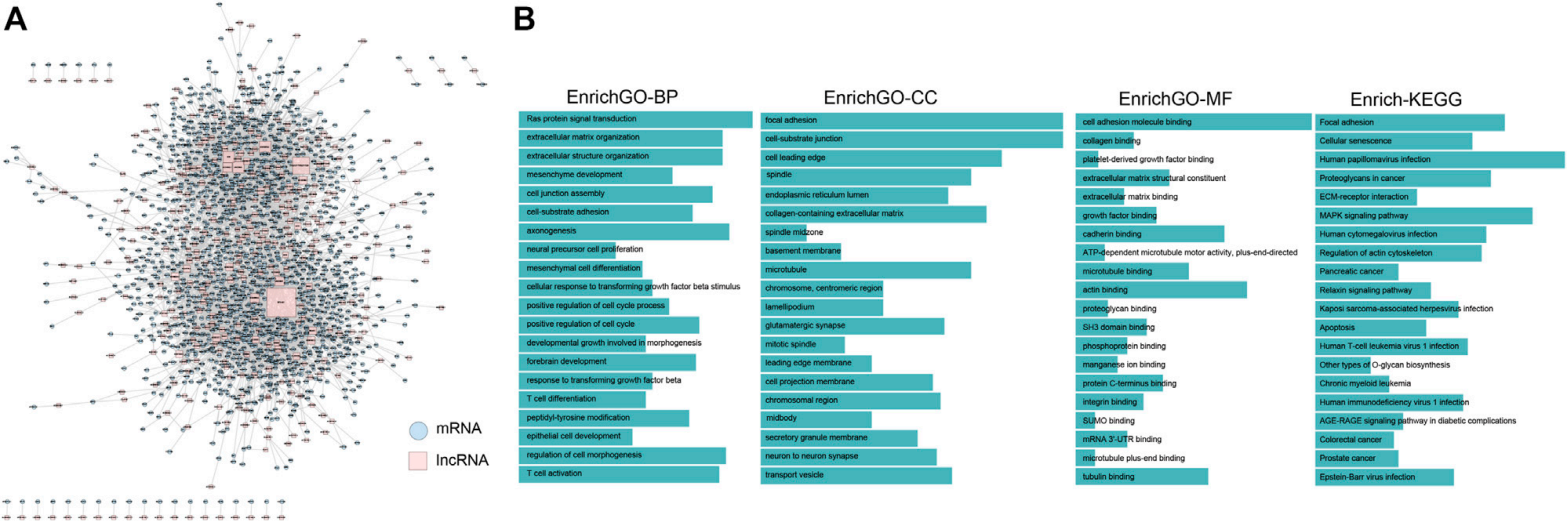
**(A)** lncRNA-mRNA ceRNA network specific to glioblastoma. **(B)** The enrichment analyses of mRNAs on the glioma-specific ceRNA network, in which GO includes BP, MF and CC.

Recent studies have reported that lncRNAs can participate in competing endogenous RNAs (ceRNAs) regulations in order to communicate with other RNA transcripts. In order to better understand the regulatory relationship between differential mRNA and lncRNA, subsequently, we mapped the resulting DElncRNAs and DEmRNAs to the lncRNA-ceRNA relationship pairs downloaded from starbase and constructed a glioblastoma-specific ceRNA network (Figure 2A) which was composed of 343 lncRNAs, 1,427 mRNAs, and a total of 3,741 edges. Gene Ontology and Kyoto Encyclopedia of Genes and Genomes pathway enrichment analyses revealed that the DElncRNAs involved in the ceRNA network were remarkably associated with a series of functions, namely, T cell activation, epidermal cell development, MAPK signaling, and cell apoptosis (Figure 2B). In addition, we counted the types of cancer associated with each lncRNA on the ceRNA network by using the lnc2Cancer database and performed functional enrichment of the interacting mRNAs *via* clusterProfiler, the results of functional enrichment analysis are listed in.

### Screening for lncRNAs Significantly Associated With Survival Involved in ceRNA Network

**FIGURE 3:**
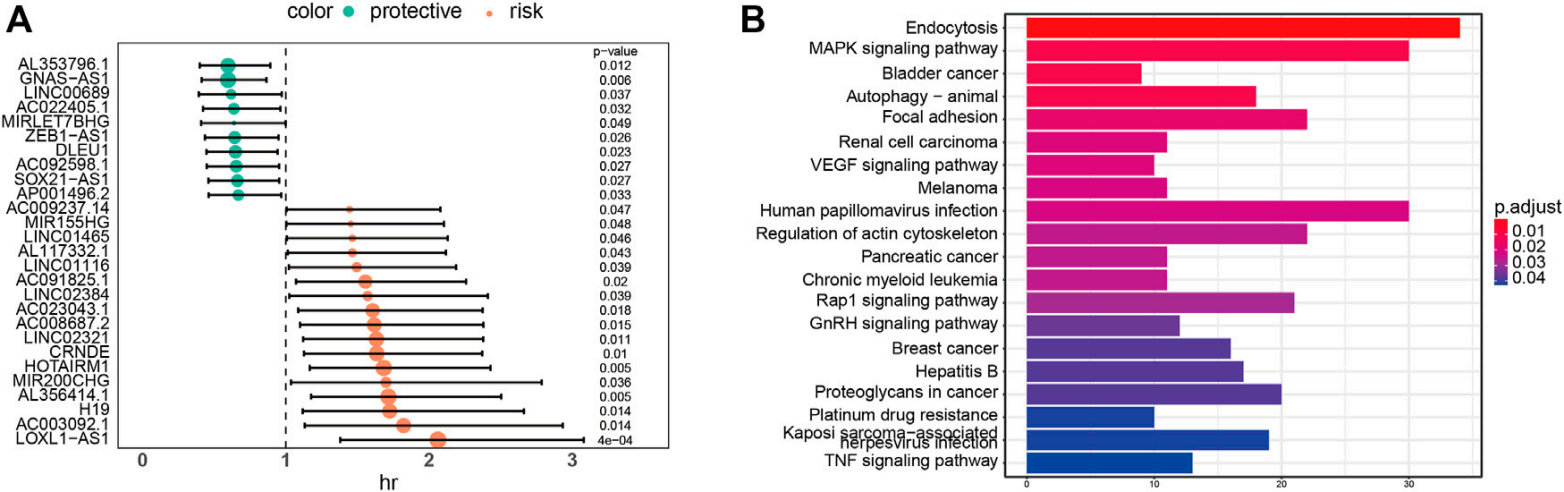
**(A)** Univariate Cox proportional hazards model was applied to identify the 27 lncRNAs significantly associated with survival. **(B)** KEGG pathway enrichment analysis of 27 lncrNA-regulated mrnas significantly associated with survival.

To further analyze the relationship between lncRNA and glioblastoma prognosis in glioblastoma-specific ceRNA networks, all lncRNAs of the ceRNA network were incorporated into the univariate Cox model to spot lncRNAs significantly associated with survival based on the lncRNA expression and clinical information. As a result, using the threshold value of *p* < 0.01, 27 lncRNAs containing LOXL1-AS1 and HOTAIRM1 were revealed to be prominently associated with GBM prognosis among 343 lncRNAs (Figure 3A). The knockdown expression of LOXL1-AS1 has a functional inhibitory effect on the proliferation of GBM cells (Wang et al., 2018), which has been confirmed in the literature. Moreover, HOTAIRM1 knockdown has also been confirmed to significantly weaken the migration and invasion of GBM cells (Xie et al., 2020). Next, functional annotations were performed on the mRNAs regulated by identified lncRNAs significantly associated with survival in the ceRNA network, and we found that the mRNAs regulated by these lncRNAs significantly enriched MAPK signaling pathways and Focal adhesion (Figure 3B). It has been confirmed that MAPK signaling pathway is significantly correlated with survival and prognosis of glioma (Nicolas et al., 2019; Chen et al., 2020). In addition, GSK2256098 is a novel oral focal adhesion kinase (FAK) inhibitor. Preclinical studies demonstrate growth inhibition in glioblastoma cell lines (Swartz, 2020). These results indicate that the 27 lncRNAs identified by us can affect the survival prognosis of glioma patients.

### Classification of Glioma Patients Based on LncRNA Related to Prognosis

**FIGURE 4:**
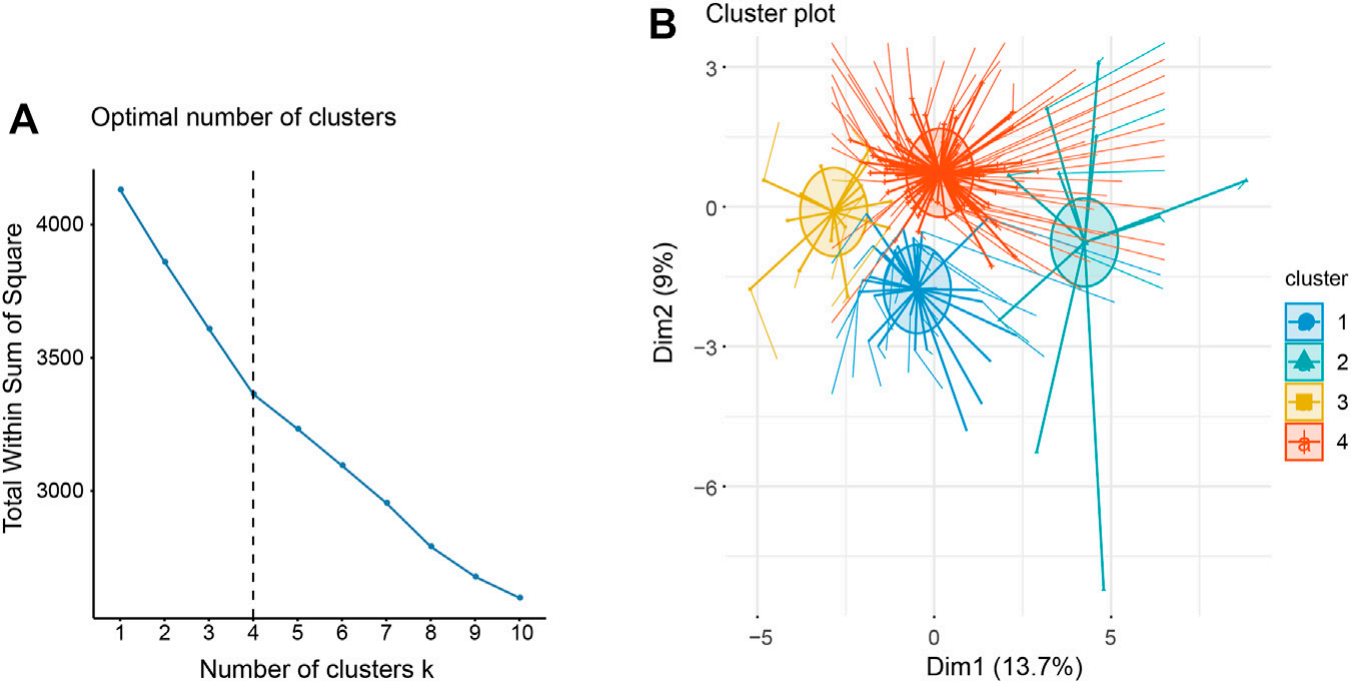
**(A)** The optimal number of clusters for K-means clustering, that is, K = 4. **(B)** Cluster graph of K-means.

After the above identification, it was known that the 27 lncRNAs related to survival can be used to judge the survival of patients. Continuously, based on the expression of 27 lncRNAs significantly related to survival, using the K-means clustering method, we gathered samples into four categories on the basis of the optimal number of clusters as k = 4 (Figures 4A,B). As results, 33 samples were divided into cluster 1, 11 samples were divided into cluster 2, and clusters 3 and 4 contained 16 and 94 samples, respectively.

### Survival Analysis for the Glioblastoma Patients of the Four Subtypes

**FIGURE 5:**
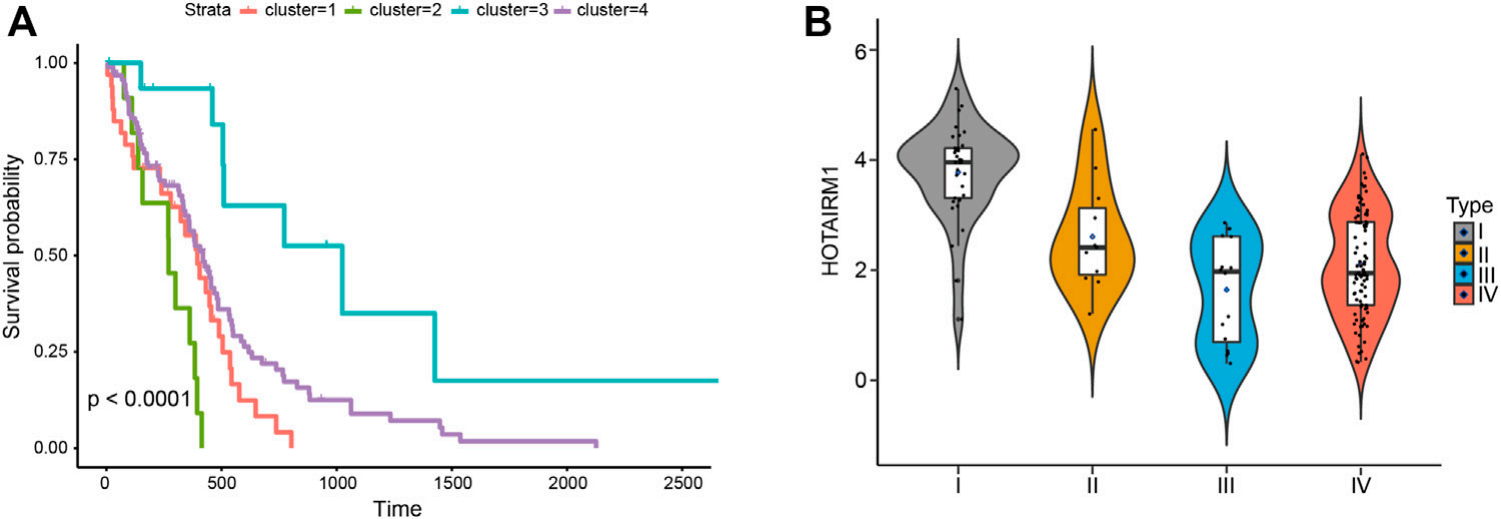
**(A)** Kaplan–Meier survival curves of four types of samples. **(B)** The violin diagram shows the expression of lncRNA HOTAIRM1 in samples of four different subtypes.

To determine lncRNAs whether have an impact on the overall survival of patients with GBM, survival analyses were conducted to investigate the Kaplan–Meier curves for GBM patients with the 27 lncRNAs (Figure 5A). The result exhibited an obvious difference in survival time between the four categories of samples (*p* < 0.05). It indicates that these 27 lncRNAs may be potential prognostic factors for glioblastoma. Then, we evaluated the differential expression of 27 lncRNAs significantly associated with survival in different subtypes. We found that lncRNA HOTAIRM1 was highly expressed in subtype I compared with the other three subtypes. And it’s been documented that Serum long noncoding RNA HOTAIR as a novel diagnostic and prognostic biomarker in glioblastoma multiforme. The higher the expression of HOTAIR, the worse the survival of patients (Tan et al., 2018). In our study, HOTAIRM1 was highly expressed in the samples of subtype 1 with the worst prognosis, while HOTAIRM1 expression was lowest in the samples of subtype 3 with a good prognosis (Figure 5B). This indicates that the lncRNAs identified by us can accurately classify patients and explain the clinical results of the corresponding subtypes.

**FIGURE 6:**
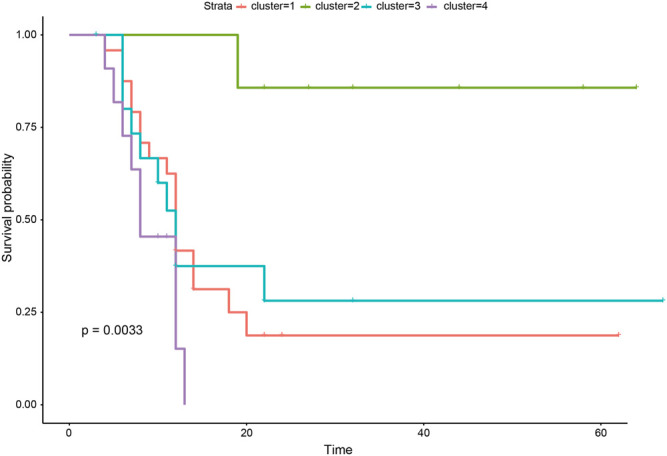
Kaplan–Meier survival curves of four types of samples in independent validation set.

### Robustness Analysis of 27 lncrnas Significantly Associated With Prognosis

To verify the accuracy of patient classification based on the identification of 27 lncRNAs significantly associated with survival, we downloaded a set of transcriptome data (GSE121720) from the Gene Expression Omnibus (GEO) database (https://www.ncbi.nlm.nih.gov/geo/) containing 60 glioma samples, and the survival time of patients was available. Similarly, we classified the samples based on the identified lncRNAs significantly associated with survival using k-means. In the independent validation set, we also divided the samples into four categories, and there were significant differences in the survival time of the four categories (Figure 6).

## Discussion

GBM is a common aggressive brain cancer which occurs in the central nervous system with a known poor prognosis and limited treatment options (Xiao et al., 2020). Searching for possible molecular mechanisms and potential biomarkers for GBM is a current urgent task (Rahaman et al., 2002). Increasing experimental evidence suggests that aberrant expression of ncRNA, including lncRNA and miRNA, are intimately associated with malignant progression and metastasis (Li et al., 2019; Liu et al., 2019). Since the ceRNA hypothesis was proposed, researchers have gained increasing interest in ceRNA networks, where lncRNA may influence mRNA transcription and expression by interacting with miRNA (Li et al., 2020). Competitive endogenous RNA (ceRNA) regulatory network has been confirmed to regulate expression based on competitive mechanisms and play a crucial part in multiple tumor pathological and physiological processes. ceRNAs are significant mechanisms by which lncRNAs regulating gene expression may exert huge influences on cancer. It has been extensively reported that the disorder of the ceRNA network is closely related to cancer progression (Salmena et al., 2011). For instance, a study showed that lncRNA ZEB1-AS1 functions as a ceRNA in BC, regulating the expression of the protein-coding gene fascin-1 via miR-200b (Gao et al., 2019). Thus, the ceRNA network might promote new tools for understanding the potential mechanisms of GBM and discovering potential new therapeutic targets. Here, relied on the RNA expression dataset, we proposed a ceRNA network by identifying significantly differentially expressed genes in normal samples and cancer samples.

The rapid development of bioinformatics methods provides methodological support for exploring high-throughput sequencing data (Zhong et al., 2021). The differential RNA expression observed in between the GBM and normal samples suggests that DERNAs may exert a critical role in cancer progression. In this study, we identified differentially expressed lncRNAs and mRNAs in GBM and normal brain tissue samples from the TCGA dataBase, and we further constructed a ceRNA network specific for glioblastoma combined with the lncRNA-ceRNA relationships attained in the starBase database. Functional enrichment analysis of the mRNA in the ceRNA network was performed to identify the notably enriched KEGG and GO terms. Based on the principle of the ceRNA network, lncRNA participates in biological processes by acting as endogenous molecular sponges that competitively bind to miRNAs and indirectly regulates the expression level of messenger RNA (mRNA). Hence, the potential functions and pathways of lncRNA may be similar to that of mRNA. The GO functional annotation mostly showed enrichment of mRNA related to several major regions, such as growth factor binding, Ras protein signal transduction, and positive regulation of cell cycle process. Moreover, several enriched pathways observed in the KEGG results have been reported in previous studies. MAPK is a key signaling pathway involved in GBM proliferation, apoptosis, migration, and infiltration (Vitucci et al., 2013). Finally, we assessed the survival time among the samples by clustering samples into four different subgroups based on K-means cluster analysis and the Kaplan–Meier survival curve showed remarkable differences in the survival time of the four categories of samples. This also indicates that the 27 selected lncRNAs that are significantly related to survival may be potential clinical prognostic factors for glioblastoma, and the mRNAs that interact with them may be potential therapeutic targets for glioblastoma.

Overall, we depicted a reliable prognostic ceRNA network using the differential lncRNAs and mRNAs involving GBM in the TCGA database and investigated the relevant clinical information. Our results provide a novel approach to discovering potential ceRNA networks in GBM, which will help to better understand the pathogenesis of GBM at the gene level and identify potential therapeutic agents for treating GBM.

## Data Availability

The original contributions presented in the study are included in the article/Supplementary Material, further inquiries can be directed to the corresponding author.
